# Developing a Novel Geriatrics Perioperative Case Conference Series

**DOI:** 10.1093/geroni/igaf122.1358

**Published:** 2025-12-31

**Authors:** Evan Henricks

**Affiliations:** Medical College of Wisconsin, Milwaukee, Wisconsin, United States

## Abstract

This session aims to demonstrate how support from career development awards enables the creation and implementation of educational interventions that improve knowledge base and change practice in medical professionals. With support from the Geriatric Academic Career Award (GACA) this project developed a novel, recurring, geriatrics perioperative case conference series. Using the Kern method of curriculum development, targeted providers (physician, advanced practice provider, and nursing) were identified and asked to complete a voluntary, anonymous self-assessment of needs, ranking interest in six different topics of age-friendly elements (4Ms) in the perioperative care of older adults. A case conference series was then created and implemented with preliminary learner competence data collected using an anonymous self-assessment survey. 24 participants (36% of sample) completed the needs assessment survey. After creation and implementation of the two of three planned case-series, 9 learners’ post-session evaluation (response rate 22%), rated level of comfort on the topic higher post-session (mean 4.11) compared to pre-session (mean 3.0) with nearly 90% of participants replying “would recommend this session” to others. All attendees were either “somewhat likely” or “extremely likely” to utilize geriatrics templates, screening tools, and recommend interventions after attending sessions. Perioperative providers are interested in learning about age-friendly health elements, specifically regarding mentation, medications, and mobility. The targeted curriculum increased the level of comfort in clinical care of older adults, with participants likely to recommend the session. Development and implementation of this curricular intervention was possible through the support of the GACA funding.

